# Forecasting life expectancy in São Paulo City, Brazil, amidst the COVID-19 pandemic

**DOI:** 10.1186/s12963-025-00397-7

**Published:** 2025-07-06

**Authors:** Maria L. Miranda, Cassio M. Turra, Ugofilippo Basellini

**Affiliations:** 1 Max Planck Institute for Demographic Research, Rostock, Germany; 2 Demography Department, CEDEPLAR, UFMG, Belo Horizonte, Brazil

**Keywords:** Mortality forecasting, Covid-19, Lee-Carter, Lee-Miller, São Paulo

## Abstract

**Background:**

The COVID-19 pandemic has significantly increased mortality rates, disrupting historical trends and making it challenging to forecast future life expectancy levels. São Paulo, the first city in Brazil to report a COVID-19 case and death, saw a decrease of over four years in life expectancy at birth for males and over three years for females between 2019 and 2021. São Paulo has been at the forefront of the demographic transition in the country and experienced a nonlinear mortality decline over the twentieth century. The city's historical mortality trajectory and the disruptive effects of COVID-19 have introduced challenges to mortality forecasting.

**Methods:**

In this study, we used a unique dataset dating 1920–2022 to forecast life expectancy in São Paulo until 2050 using the Lee-Carter and Lee-Miller methods. Mortality rates were obtained from a combination of deaths gathered by the SEADE Foundation (SEADE) and population collected by the Brazilian Institute of Geography and Statistics. To mitigate the dependency on the fitting period's choice and better incorporate the effects of the recent mortality shock, we used different baseline periods, using all years from 1920 to 1995 as the starting year of the analysis and six scenarios for post-pandemic mortality levels. Prediction intervals were derived from simulated trajectories of the models' time indices. Based on 73,200 simulations for each year between 2023 and 2050, we synthesized the resulting life expectancy forecasts into median values and 95% prediction intervals (PI).

**Results:**

By 2050, we predict that life expectancy at birth in São Paulo will reach approximately 81.4 years for men and 88.3 years for women. Also, within the 95% PI, we estimated that by 2045, male life expectancy could reach the levels of best-performing countries.

**Conclusions:**

Our approach is among the first attempts to forecast mortality in the presence of shocks. Additionally, by evaluating different baseline periods, we advocate for the adoption of more accurate forecasting strategies, particularly in contexts of recent mortality decline. These findings provide valuable resources for policymakers and researchers working to address public health challenges arising from the pandemic and plan for the future well-being of many populations.

**Supplementary Information:**

The online version contains supplementary material available at 10.1186/s12963-025-00397-7.

## Background

São Paulo has been at the forefront of the demographic transition in Brazil. Over the twentieth century, the health transition has evolved in the city, improving survival levels and changing mortality patterns [1]. Improvements in public health through the control of diseases, the provision of water, sanitation, and health services, and the combat of violence reduced mortality differently across age groups in the past century, resulting in a non-linear pace of mortality decline [2]. More recently, the effects of COVID-19 in 2020–2022 added more complexity to the historical trajectories of the health transition.

The COVID-19 pandemic triggered an unprecedented rise in mortality globally over the past few years. Such impact was significant enough to change historical trends, resulting in reduced life expectancy and a surprising number of excess deaths for some populations. The discrepancies across countries were mainly due to differences in the age structure [3] and context-specific factors, including socioeconomic conditions, chronic disease prevalence, and public policies to combat the virus spread [4].

Estimates show declines of more than four years in life expectancy at birth between 2019 and 2021 in Bolivia, Botswana, Lebanon, Mexico, Oman, and the Russian Federation [5]. Similar analyses show substantial life expectancy reductions in Eastern Europe and the United States between 2019 and 2021, but not among the Western European countries [6].

In Brazil, the estimated reduction in life expectancy was 1.3 years between 2019 and 2020 [7]. Until May 2021, Brazil was considered the second country with the most notable excess deaths after COVID-19 [8]. From March 15 to June 6, 2020, there were over 62,000 excess deaths caused by diseases observed, representing a 22% increase in overall mortality over 12 weeks [9]. Due to the concentration of the population in major cities, the impact of the pandemic on mortality was more pronounced in urban areas, particularly in the capital cities. São Paulo city, the state's capital, recorded a disproportionately higher number of excess deaths, accounting for 36% (6,208) above the expected average from March 15 to June 6, 2020 [9]. By December 2022, the city had confirmed over 1.1 million cases and more than 44,000 deaths due to COVID-19 [10].

Nevertheless, estimating the pandemic's impact on mortality remains challenging, even when considering measures such as excess mortality, which mitigates the effect of misidentification of COVID-19 as the underlying cause of death. Age-specific case counts depend highly on the testing capacity, testing strategy, and differences in the definition of cases across sources and over time, especially in low- and middle-income countries [11]. Also, due to the behavioral changes induced by the pandemic and the differential fatality rates associated with the presence of comorbidities, establishing a causal relationship between COVID-19 and other causes of death occurring after 2020 becomes complex [12]. It is possible that the rates of these chronic ailments may have or will decrease if patients succumb to COVID-19, a process known as the "harvesting effect" [12, 13]. Also, due to the reduced social and work activities, deaths from specific external causes may have declined during the pandemic [12]. Conversely, deaths from other causes may have increased due to several aspects, including inadequate care in clinics and hospitals because of shortages of equipment, staff, and space, worsening of comorbidities owing to the effects of COVID-19, and other factors [14].

In summary, it is challenging to assess the impact of COVID-19 on mortality due to its interaction with other causes of death [12] and data issues [11]. Additionally, it is uncertain when mortality levels will return to pre-pandemic levels. Despite the uneven distribution of healthcare infrastructure and ongoing cuts in health spending in Brazil, the public health system has remained operational during the COVID-19 pandemic [15]. In January 2021, the vaccination campaign started in the country, with São Paulo being the first city to vaccinate. The campaign encountered several challenges, including political instability, lack of national planning, delays in securing supplies such as syringes and needles, and vaccine shortages [15, 16]. However, given Brazil's historical culture of immunization, more than 80% of Brazilians aged six months and older completed the primary vaccination schedule as of 2022 [17]. This high acceptance of immunization among the population led to a large reduction of COVID-19 deaths in 2022 (75,429) compared to 2021 (427,629), despite the similar number of identified cases in the two years: 14,768,820 in 2021 and 14,096,655 in 2022 [18]. Considering this rapid decline in COVID-19 deaths, it is plausible that mortality levels will return to pre-pandemic levels in the coming years. However, there remains uncertainty regarding the mortality level's return.

Estimating and forecasting mortality tendencies is essential for designing public policies, assessing health insurance premiums, and examining the solvency of pension systems. It is also a way to indicate levels of well-being across nations, regions, and other population subgroups. However, to estimate future trends, it is necessary to have robust enough historical data to extrapolate mortality levels for the following decades. Additionally, forecasts must consider the uncertainty caused by variations in the observed trend, such as mortality shocks and non-linear declines. São Paulo may be the only place in Brazil where a comprehensive historical series of age-specific mortality rates by sex has been documented, tracing back to 1920. The data quality of this historical series has been examined before and proved reliable enough to allow demographic exercises, even though it is not error-free [19–21].

São Paulo is recognized for the reliability of its demographic data within the Latin American context, particularly in terms of accurate death registration and population enumeration. However, to ensure data consistency, we applied corrections for the under-registration of death until the year 2000. In addition, following the methodology proposed by Berquó [22], we excluded estimated deaths of non-residents from the records prior to 1969. Similar datasets and correction approaches have been used in previous studies, such as Silva [20] and Siviero [21], which strengthens the robustness of our data adjustments.

In this study, we estimate future life expectancies in São Paulo city until 2050, incorporating the uncertainty caused by COVID-19 and non-linear changes in mortality trends over the last century. To do this, we use Lee-Carter [23] and Lee-Miller [24] methods with different base periods, starting between 1920 and 1995 until 2022, and six scenarios for post-pandemic mortality levels. Death rates were obtained from the combination of deaths gathered by the SEADE foundation (SEADE) and population collected by the Brazilian Institute of Geography and Statistics (IBGE) data.

## Methods

The Lee-Carter (LC) is a stochastic method developed by Ronald D. Lee and Lawrence R. Carter in 1992 to model and forecast age-specific death rates, combining demographic mortality and time series models [23]. The model was proposed as follows:

$$\text{ln}\left({m}_{x,t}\right)= {a}_{x}+{b}_{x}{k}_{t}+{\varepsilon }_{x,t}$$

where $\text{ln}({m}_{x,t})$ denotes the logarithm of the death rates by age group x at year t; ${a}_{x}$ describes the average shape of the mortality by age; ${b}_{x}$ describes the extent to which mortality at age x changes given the overall temporal change in the general level of mortality; ${k}_{t}$ is the mortality level index at time t; and ${\varepsilon }_{x,t}$ is an error term reflecting residual age-specific influences not captured by the model.

In the original version, Lee and Carter [23] proposed an adjustment on the ${k}_{t}$ parameter, so the number of deaths estimated by the method using fixed ${a}_{x}$ and ${b}_{x}$ would match the ones observed each year t. After this adjustment, they proposed forecasting the ${k}_{t}$ s using a random walk process with drift. This time series model can be expressed as follows:

$${k}_{t}=c+{k}_{t-1}+{u}_{t}$$

where $c$ is the drift and ${u}_{t}$ is a white noise process with mean zero and variance ${\sigma }^{2}$.

Lee and Carter [23] introduced an innovative and straightforward approach to forecasting mortality rates, yet the method has limitations and disadvantages that have led to the development of new variants. Lee and Miller proposed one of them in 2001 (LM) to address some of the main limitations of the LC, specifically: (i) the jump-off error, (ii) the assumption of fixed changes in the mortality pattern over time, and (iii) the adjustment of the time index ${\widehat{k}}_{t}$ [24].

Other variants also tried to account for different limitations of the model in the demographic and actuarial literature. For instance, Booth et al. [25] proposed modifications to the LC method to adjust the time component to reproduce the age distribution of deaths rather than the total number of deaths, and to determine the optimal fitting period to account for nonlinearity in the time component. Renshaw and Haberman [26] extended the model to account for cohort effects, while Hyndman and Ullah [27] suggested using the functional data paradigm [28] to capture additional dimensions of change in mortality rates from the log death rates. Li et al. [29] proposed using a rotation in the projected age pattern of mortality decline to account for the observed changes in the ${b}_{x}$ over time. Additional extensions of the original method can be found in the recent literature review by Basellini et al. [30].

Forecasting models that account for shocks have been extensively studied in other fields, including econometrics and macroeconomics. Traditional time series models, such as those introduced by Box and Jenkins [31], provide a basic framework for forecasting, but often struggle to account for structural breaks or regime shifts. To address these challenges, alternative approaches have been developed, including vector autoregressions (VAR) [32, 33] and structural break models [34, 35], which explicitly incorporate abrupt changes in time series dynamics.

In addition, regime-switching models, such as those proposed by Hamilton [36] and further refined by Kim and Halbert [37], allow for distinct phases in time series behavior, making them particularly useful when shocks induce persistent changes rather than temporary fluctuations. Finally, a common strategy in econometrics involves the use of dummy variables to capture the effects of specific shocks, allowing for adjustments in model estimates before and after the event [38, 39]. While these methods have been widely used in macroeconomic forecasting [40, 41], they also offer valuable insights for demographic forecasting, particularly for capturing the long-term effects of unprecedented events such as pandemics or economic crises on mortality trends.

In this work, we apply the LC and LM methods to forecast life expectancy at birth in São Paulo city until 2050. These models were chosen due to their simplicity and widespread acceptance in mortality forecasting. All estimated parameters and adjustments were made in accordance with the proposals of the authors for both methods.

*Starting points* There has been a significant change in living conditions in São Paulo over the last century. This has resulted in non-linear changes in the city’s mortality pattern [2]. When it comes to forecasting, historical trends are usually extrapolated into the future, including the Lee-Carter [23] and Lee-Miller [24] methods. However, this can lead to considerably different forecasted results depending on the chosen baseline period, especially if there are non-linear changes in the mortality level over time. To address this issue, Lee and Miller [24] suggested using data after 1950 to better meet the assumption of fixed ${b}_{x}$. Similarly, Hyndman and Booth [42] adopted the starting year of 1950 to avoid external influences in the mortality trend, such as the two world wars and the Spanish Influenza in 1918, as well as structural changes over the last century.

The choice of using 1950 as a starting point is subjective and may not be applicable to all countries. While some studies such as those by Lee and Miller [24], Tuljapurkar et al. [43], and Hyndman and Booth [42] suggest that 1950 is a suitable starting point, it is uncertain whether this year accurately represents mortality patterns in less developed countries. For instance, in Brazil, mortality rates began to decrease after 1940 [1], and there were non-linear declines throughout the last century, resulting in varying rates of increase in life expectancy at birth [2].

This study used different starting points to forecast life expectancy until 2050, aiming to prevent the subjectivity of choosing periods with fast or slow mortality declines. The approach was to use baseline periods starting at each year between 1920 and 1995 to forecast life expectancy in São Paulo city from 2023 to 2050. For the LM method, baseline periods started between 1950 and 1995. As such, for each COVID-19 scenario and choice of the final year of the fitting period (which will be presented in the next subsection), we run the LC method for 76 different baseline periods and the LM for 46, separating men and women.

*Scenarios for COVID-19* The COVID-19 pandemic led to a significant increase in deaths, particularly in Brazil, which had the second-highest number of excess deaths after COVID-19 [8]. It's uncertain when mortality levels will return to pre-pandemic levels despite the fast decline in COVID-19 deaths and the widespread acceptance of vaccinations among the population [17]. To address this issue, we propose using six different scenarios for the recovery of mortality levels in the years following COVID-19. These scenarios were computed by including or not the observed death rates of the three years of COVID-19 (2020, 2021 and 2022) and the creation of assumptions for the years after 2022.

The first scenario utilized data until 2019 and assumed that the mortality level observed before the pandemic would continue in the following years. The second scenario used data until 2022, factoring in the pandemic's impact on mortality rates over the past three years. The third scenario considered the three years of COVID-19 and a hypothetical year of 2023, assuming that mortality levels would return to pre-pandemic rates (i.e., 2019). Moreover, the fourth scenario also included the three years of COVID-19 and a hypothetical year of 2023, basing mortality levels on the forecasted levels from the first scenario.

Considering the challenges faced during the COVID-19 vaccination campaign in Brazil, which began in 2021 [15, 16], it is likely that the effects of the pandemic may only be fully realized in 2024. To explore this further, we have applied the methodology used by the United Nations in their World Population Prospects [5] to two alternative scenarios, where mortality levels return to pre-pandemic levels in 2024 instead of 2023. Scenario five assumes three years of COVID-19 (2020, 2021 and 2022), followed by a hypothetical year in 2024 with mortality levels equal to those observed in 2019, and a hypothetical year in 2023 with mortality levels between those of 2022 and 2024. In this scenario, it is assumed that mortality levels will return to pre-pandemic levels in 2024, with 2023 being an intermediary year. Scenario six also assumes three COVID-19 years (2020, 2021 and 2022), but with a hypothetical year in 2024 based on the forecasted year from scenario one (following the same starting year iteration), and a hypothetical year in 2023 with mortality levels between those of 2022 and 2024. In this scenario, it is assumed that mortality levels will return to the expected levels for 2024 amidst the pandemic, with 2023 being an intermediary year. Finally, we recall here that for each of these six scenarios, we employ a variety of starting years for the fitting period (see previous subsection), resulting in 456 (76*6) different fitting periods for the LC method and 276 (46*6) fitting periods for the LM.

**Fig. 1:**
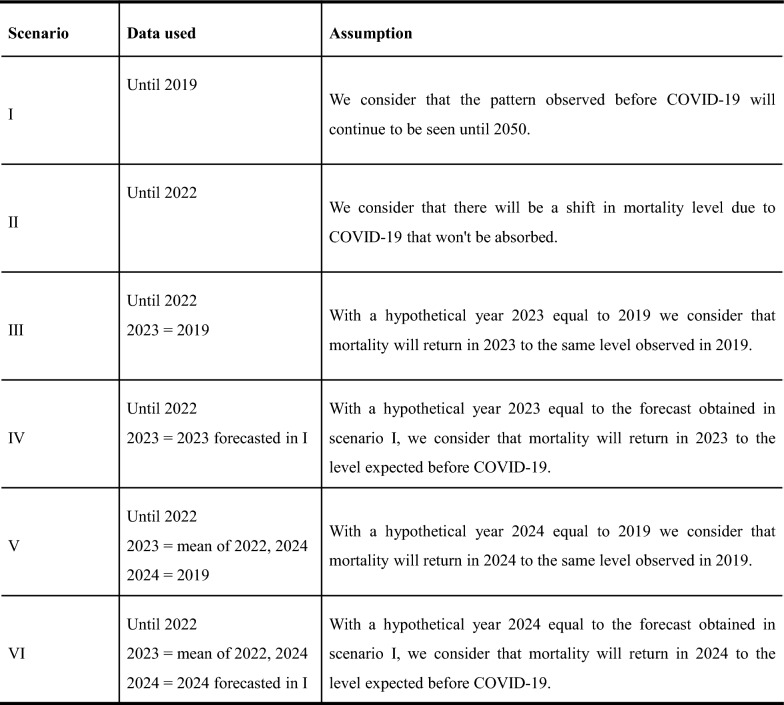
Summary of the six scenarios considered for the return of mortality levels after the pandemic

Figure 1 summarizes all six scenarios. It is important to note that other mortality outcomes may occur in São Paulo city in the next years. However, by including the six scenarios into the forecasts, it was possible to account for some of the uncertainty regarding the mortality level's return post-pandemic.

*Final estimate and prediction intervals* To compute prediction intervals for life expectancy, we use a simulation approach for the forecasted ${k}_{t}$ s. Specifically, we simulated the forecasted ${k}_{t}$ s 100 times and computed the life expectancy at birth for each simulation over the forecast horizon (2023–2050). This resulted in a total of 45,600 forecasts of life expectancy at birth between 2023 and 2050 for the LC method and 27,600 forecasts for the LM method for men and women separately. From these estimates, we obtained the median and the 2.5 and 97.5 quantiles. The quantiles allowed us to compute the 95% prediction interval for each forecast year, with the median used as the final estimate. We note here that this approach may under- or (more likely) over-estimate the width of the prediction intervals, as it amounts to treating the simulations from the different fitting periods as independent between each other.

Our final approach considered different baseline periods, six scenarios of survival recovery after the pandemic, and 100 simulations of the time-index parameter, allowing us to better account for the forecast uncertainty in the context of rapid changes in mortality levels. However, it's important to note that our analysis does not account for other potential mortality outcomes that may occur in São Paulo city in the coming years. The effect of the pandemic on mortality and other changes in mortality patterns induced by future shocks are yet to be predicted.

## Results

**Fig. 2:**
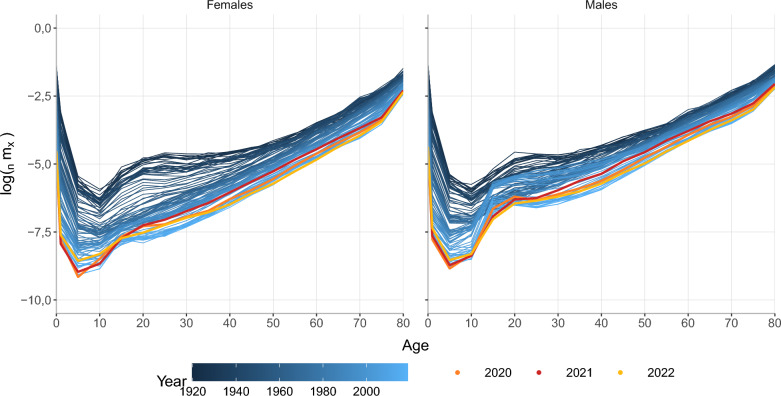
Log of age-specific death rates by sex, São Paulo city 1920–2022

Figure 2 shows the logarithm of age-specific death rates by sex for São Paulo from 1920 to 2022. The figure reveals a consistent mortality decline over the years, with the highest level observed in 1920. Comparing the age groups, the most considerable improvement in mortality, especially among females, occurred at younger ages (up to age 50). Our results align with previous research, which has consistently shown higher mortality rates among men than women. Also, the age patterns follow previous studies [44, 45], with decreases over time that led to a structure of higher mortality rates at birth that decreases until around age ten and increases again with a hump between ages 15–30. This peak, more pronounced among men, is attributed to external causes of death, such as accidents and violence, which have become more prevalent in Brazil over the last decades, affecting especially men at young ages [46].

Figure 2 highlights the years 2020, 2021, and 2022 in orange, red, and yellow, respectively, to indicate the period affected by COVID-19. The pandemic disrupted the pattern of decreasing death rates, causing a significant increase in mortality, especially for those above 20 years old. It is evident that the year 2021 had a more substantial impact on mortality than 2020, with a higher number of reported deaths. However, with the start of vaccination in 2021, there was a subsequent decline in the death rates in 2022. It is highly likely that mortality will revert to pre-pandemic levels in 2023 or the following years. Regarding the age pattern of mortality, the pandemic changed the hump: the peak shifted to older age groups, reflecting variations in fatality rates across different ages, particularly the concentration of fatalities among older individuals [47]. After 2021, the hump seems to shift back to younger age groups.

**Fig. 3:**
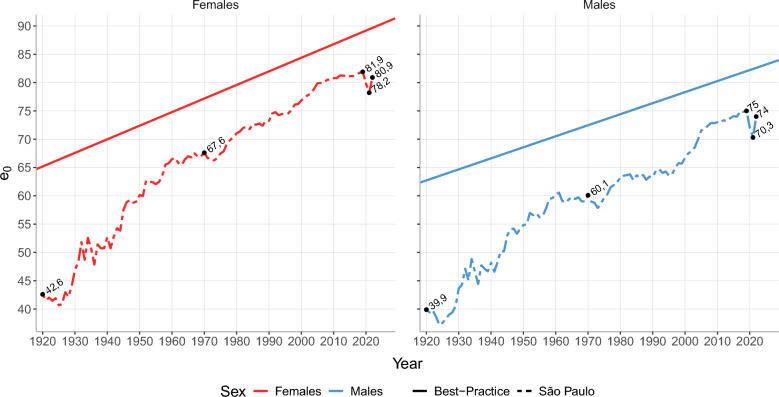
Life expectancy at birth by sex, São Paulo city 1920–2022 and the Best-Practice slope

Figure 3 reveals that life expectancy for both men and women has improved significantly since 1920. In that year, the level was around 40 and 43 years for men and women, respectively, whereas in 2019, it was close to 76 and 83 years. Nevertheless, over this period, life expectancy at birth did not follow a linear trend, followed by a non-expected drop after 2019 due to the COVID-19 pandemic. Between 2019 and 2021, life expectancy at birth has dropped by more than four years for males and more than three years for females. In 2022, however, life expectancy levels bounced back, returning to similar levels as of 2019. By 2022, male and female life expectancy at birth is estimated to be around 74 and 81 years old, respectively.

São Paulo presented a complex and multifaceted mortality decline in the past century, with differences by sex and age groups. After 2000, life expectancy at birth for women in the city was already close to the estimated levels of the Best-Practice countries for men by Oeppen and Vaupel [48]. This result expresses the relatively fast convergence of mortality levels to the ones in the wealthiest countries, and it is in line with results by Vallin and Meslé [49]. By 2019, the difference between São Paulo and Best-Practice life expectancy at birth was around six years for females and seven years for males.

We analyzed mortality trends up to 2022 and used the Lee-Carter [23] and Lee-Miller [24] methods to forecast life expectancy in São Paulo city until 2050. Our methodology which included accounting for different baseline periods and scenarios for the return of mortality levels after COVID-19, led to 73,200 estimates of life expectancy at birth for each year between 2023 and 2050. We combined the results into median and 95% prediction intervals for both males and females.

**Fig. 4:**
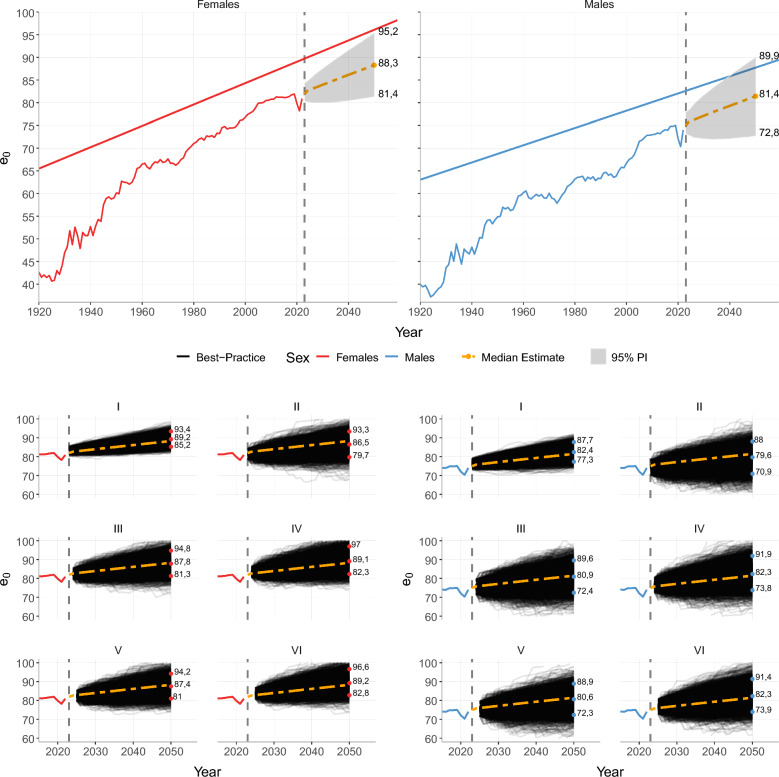
Life expectancy at birth forecasts with 95% prediction intervals until 2050 (top) forecasts by each scenario (bottom), São Paulo city

Figure 4 shows life expectancy forecasts by sex. The results for females are displayed in the left panel. The median forecast for 2023 is 81.9, with a 95% PI ranging from 80.5 to 84.2. The estimated increase for this year is enough to reach the pre-pandemic level. For 2024, the median estimate is 82.6, and the 95% PI ranges from 80.3 to 84.6. From 2024 onwards, there is a steady increase in life expectancy until 2050, with estimated life expectancy at birth equal to 88.3 and 95% PI between 81.4 and 95.2.

The prediction intervals for females are relatively narrow, indicating more undersized uncertainty in the forecasts. This is likely due to the more consistent decrease in mortality over the last century and the shorter impact of COVID-19 on female mortality. The widest 95% prediction interval is estimated in 2050, with a range of approximately 14 years.

The right panel shows forecasts for male life expectancies. The median forecast for 2023 is 75, with a 95% PI between 73.3 and 77.8. Although this is higher than the life expectancy observed in 2022 it is somewhat below the pre-pandemic level. For 2024, the median estimate of 75.8 is slightly above the level seen in 2019, with a 95% PI ranging from 73 to 78.2. From 2024 onwards, the estimates show a relatively stable increase until 2050, with estimated life expectancy at birth of 81.4 and a 95% PI ranging from 72.8 to 89.9.

It is essential to note that the prediction intervals are quite broad compared to females. This expresses the uncertainty surrounding the life expectancy levels when the different baseline periods and levels of mortality post-pandemic are accounted for in the forecasts. Additionally, it reflects the more disruptive impact of the pandemic on male mortality levels. Depending on the chosen baseline period and COVID-19 scenario, results with differences of up to 17 years in 2050 can be found within the 95% range.

It should be noted here that our forecasts—despite the occurrence of COVID-19—are significantly higher than those that have been made in the past. Specifically, we forecast that life expectancy at birth in 2050 for males and females will be 2.2 and 3.6 years higher, respectively, then what was predicted by the most recent forecast in 2013 [10].

Also, within the 95% PI of our forecasts, both male and female São Paulo life expectancy could be reaching the Oeppen-Vaupel Best-Practice [48] after 2050. For this analysis, we assumed that the Best-Practice increase will follow the same pace of increase until 2050. Additional file 1 summarizes all median estimates and 95% prediction intervals for males and females between 2023 and 2050.

## Discussion

The aim of this work was to forecast life expectancy at birth in São Paulo city until 2050 while considering the impact of the COVID-19 pandemic and the non-linear changes in mortality trends that have occurred over the past century. São Paulo has played a pioneering role in managing the pandemic in Brazil, and it also has a comprehensive historical record of age-specific mortality rates dating back to 1920. Therefore, using death rates for São Paulo was ideal for applying forecasting methods to analyze future survival levels in Brazil.

We used the LC and LM methods with death rates obtained from the combination of deaths gathered by the SEADE foundation (SEADE) and population collected by the Brazilian Institute of Geography and Statistics (IBGE) data. These methodologies have gained popularity among demographers due to their innovative and straightforward approach to mortality forecasting, although they are not without limitations. Most forecasting methods, including the LC and the LM, are extrapolative. Therefore, these methods are sensitive to the choice of the baseline period and fluctuations in the rate of mortality decline in the past.

As emphasized in our study, from 1920 to 2019, life expectancy at birth in São Paulo increased non-linearly. Moreover, there was a substantial reduction in life expectancy during the years of the pandemic. To mitigate the dependency on the fitting period's choice and better incorporate the effects of the recent mortality shock caused by COVID-19, we used different baseline periods, from 1920 and 1995 until 2022, and six scenarios for post-pandemic mortality levels. Also, we simulated the forecasted time-index parameters to compute prediction intervals.

Based on 73,200 simulations for each year between 2023 and 2050, we summarized the resulting life expectancy forecasts into median values and 95% prediction intervals. We are unaware of any estimates that have accounted for pandemic effects and explored the non-linear changes in mortality patterns over the past century in São Paulo. Also, adding recent data enhanced the forecasts by considering the city's most recent mortality experiences. Despite the negative effects of the COVID-19 pandemic, our results presented significantly higher estimates than predicted by the most recent forecast in 2013 [10]. By 2050, we estimate that life expectancy at birth in São Paulo city will reach approximately 81.4 (95% PI ranging from 72.8 to 89.9) for men and 88.3 (95% PI ranging from 81.4 to 95.2) for women. Also, within the 95% PI, we estimated that after 2050, male and female life expectancy could reach the Best-Practice levels established by Oeppen and Vaupel [48], extrapolated for the future.

Our forecasting approach—based on a combination of different fitting periods and future scenarios—is among the first attempts to forecast mortality in the presence of shocks. We first proposed this approach in 2023 [50], and our forecast for 2022 was extremely close to the subsequent observed data—80.9 years observed versus 80.9 years forecasted for females and 74 years observed versus 73.6 years forecasted for males. However, the long-term effects of the pandemic on health and mortality remain unclear. Although vaccination rates are increasing in São Paulo city, the worsening of the interactions of COVID-19 with other illnesses, such as influenza, or the occurrence of new variants is yet to be predicted. Therefore, it is essential to note that different mortality outcomes—not accounted for in our six scenarios—may occur in the city in the coming years.

Finally, by including different baseline periods, starting from 1920 to 1995 until 2022, we were able to combine forecasts obtained over different estimated ${b}_{x}$ s, one for each baseline period, and consequently, account for some possible changes in the age pattern over time. Future research could, however, focus on applying methods that account for variations in the ${b}_{x}$, such as Li, Lee, and Gerland [29], or methodologies that are not based on the Lee-Carter framework. Furthermore, future work should be directed to quantify the correlation between forecasts derived from different fitting periods and its impact on the width of prediction intervals.

## Conclusions

In conclusion, this study provides a comprehensive analysis of future life expectancy in São Paulo city, considering the significant impact of COVID-19 and the non-linear changes in mortality trends observed over the past century. Using different baseline periods and post-pandemic scenarios allows for a more nuanced understanding of the potential trajectories of life expectancy, reflecting both historical and recent mortality patterns. Our findings indicate a substantial recovery in life expectancy post-pandemic in the short-term, and significant increases for both men and women by 2050.

The analysis and forecasts presented in this study provide a critical resource for policymakers and researchers focused on addressing public health challenges arising from the pandemic and improving population well-being. However, our results share the same limitations as other extrapolation methods. Historical patterns may not hold in the future, and unpredicted structural changes, such as new mortality shocks, may occur.

## Supplementary Information

Additional file1. Table S1. Summary of life expectancy estimates and 95% prediction intervals by sex, São Paulo city 2023–2050

## Data Availability

Data is available upon request. R codes are available at the open-access GitHub repository https://github.com/marialaura-mllm/ForeCOVID_SP.

## Abbreviations

IBGE: Brazilian Institute of Geography and Statistics (Instituto Brasileiro de Geografia e Estatística)
HMD: Human Mortality Database
LC: Lee-Carter method
LM: Lee-Miller method
SEADE: SEADE foundation (Fundação SEADE)
